# Towards Standardization for Determining Dissolution Kinetics of Nanomaterials in Natural Aquatic Environments: Continuous Flow Dissolution of Ag Nanoparticles

**DOI:** 10.3390/nano12030519

**Published:** 2022-02-02

**Authors:** Lucie Stetten, Aiga Mackevica, Nathalie Tepe, Thilo Hofmann, Frank von der Kammer

**Affiliations:** Environmental Geosciences, Centre for Microbiology and Environmental Systems Science, University of Vienna, Althanstrasse 14, UZA II, 1090 Vienna, Austria; lucie.stetten@univie.ac.at (L.S.); aima@env.dtu.dk (A.M.); nathalie.tepe@univie.ac.at (N.T.); thilo.hofmann@univie.ac.at (T.H.)

**Keywords:** engineered nanomaterials, flow-through dissolution testing, aquatic environments, OECD guidelines, environmental risk assessment

## Abstract

The dissolution of metal-based engineered nanomaterials (ENMs) in aquatic environments is an important mechanism governing the release of toxic dissolved metals. For the registration of ENMs at regulatory bodies such as REACH, their dissolution behavior must therefore be assessed using standardized experimental approaches. To date, there are no standardized procedures for dissolution testing of ENMs in environmentally relevant aquatic media, and the Organisation for Economic Co-operation and Development (OECD) strongly encourages their development into test guidelines. According to a survey of surface water hydrochemistry, we propose to use media with low concentrations of Ca^2+^ and Mg^2+^ for a better simulation of the ionic background of surface waters, at pH values representing acidic (5 < pH < 6) and near-neutral/alkaline (7 < pH < 8) waters. We evaluated a continuous flow setup adapted to expose small amounts of ENMs to aqueous media, to mimic ENMs in surface waters. For this purpose, silver nanoparticles (Ag NPs) were used as model for soluble metal-bearing ENMs. Ag NPs were deposited onto a 10 kg.mol^−1^ membrane through the injection of 500 µL of a 5 mg.L^−1^ or 20 mg.L^−1^ Ag NP dispersion, in order to expose only a few micrograms of Ag NPs to the aqueous media. The dissolution rate of Ag NPs in 10 mM NaNO_3_ was more than two times higher for ~2 µg compared with ~8 µg of Ag NPs deposited onto the membrane, emphasizing the importance of evaluating the dissolution of ENMs at low concentrations in order to keep a realistic scenario. Dissolution rates of Ag NPs in artificial waters (2 mM Ca(NO_3_)_2_, 0.5 mM MgSO_4_, 0–5 mM NaHCO_3_) were also determined, proving the feasibility of the test using environmentally relevant media. In view of the current lack of harmonized methods, this work encourages the standardization of continuous flow dissolution methods toward OECD guidelines focused on natural aquatic environments, for systematic comparisons of nanomaterials and adapted risk assessments.

## 1. Introduction

The benefits related to the specific properties of engineered nanomaterials (ENMs) have led to an increase in their use and production, raising particular attention to their environmental behavior and toxicological impact. The wide disparity in estimates of industrial production of ENMs demonstrates an important uncertainty in the quantification of ENM discharges into the environment [1,2,3]. Nevertheless, early studies have predicted that both terrestrial and aquatic environments are likely to be exposed to ENMs [1,4]. In the case of European surface waters, modeled concentrations of nanoparticles (NPs) were found to exceed 0.18 ng.L^−1^ and 150 ng.L^−1^ for Ag and ZnO NPs, respectively, in the 10% most exposed rivers, likely corresponding to rivers located near large cities and downstream river systems [5]. ENMs can be transformed before entering the environment, for instance if they are released from wastewaters treatment plants [6]. Moreover, the discharge of pristine ENMs in natural environments should also be considered, since ENMs can be released through accidental events, weathering, or directly from the use of consumer products [7,8,9,10]. 

### 1.1. Dissolution of Nanoparticles in Aquatic Environments 

Dissolution is emphasized as one of the most important mechanisms governing the fate of ENMs in aquatic environments. The oxidative dissolution of Ag NPs has been largely studied in aquatic media [11,12,13,14,15,16,17,18]. For example, Li and Lenhart, (2012) [18] showed that the dissolution of Ag NPs and the release of Ag^+^ is significantly limited in natural river waters. It has been argued that aggregation of Ag NPs may hamper dissolution due to a substantial decrease in the specific surface area [19]. However, several studies found that dissolution of Ag NPs is dependent on the particle size rather than aggregation [14,20]. For regulatory testing of ENMs dissolution rates, this information is important since up to now it was seen as a requirement to bring ENMs into a stable dispersion for dissolution testing to avoid effects of agglomeration. Extrinsic parameters such as pH, ionic strength, inorganic/organic ligands, and temperature play also an important role. Gondikas et al. [21] demonstrated that dissolution of citrate- and PVP-capped Ag NPs increases in the presence of cysteine. In contrast, several studies showed that Ag NP dissolution is inhibited in the presence of humic and fulvic acids or in the presence of natural organic matter (NOM) with high sulfur and nitrogen content [17,22]. This points to a more complex process of ENMs dissolution than only the complexation of the released cations by NOM, lowering the free ion activity and thereby enhancing the apparent solubility and dissolution rate. Finally, pH is also an important factor controlling the dissolution of Ag NPs, being enhanced at low pH values [23,24,25]. 

For soluble ENMs such as Ag NPs, it has been established that the aqueous forms (e.g., dissolved ions or aqueous complexes) are to a large extent causing the toxic effects exerted by these ENMs [26,27,28,29]. Aqueous solubility and dissolution kinetics are thus essential for (eco-)toxicological hazard testing and risk assessment strategies and are a requested information for registration of ENMs at regulatory bodies such as the European Chemicals Agency (ECHA) [30]. Determining the dissolution rates of ENMs is particularly important in determining risk/hazard since the rate of release of toxic ions prior to interaction with ligands may be more important than equilibrium concentrations, and the lifetime of the particulate form will determine the exposure to this ENM. However, high degree of uncertainty remains when it comes to understanding dissolution kinetics of ENMs in aquatic environments, mostly due to the lack of studies using aqueous media representing the heterogeneity of the world’s aquatic environments, and adapted experimental setups. 

### 1.2. Experimental Methods to Study the Dissolution of Nanoparticles in Aquatic Environments

When it comes to understanding the fate of ENMs in aquatic environments, a limiting factor is the lack of standardized experimental procedures to study the specific endpoints (solubility, dissolution rate, chemical transformation, hetero-agglomeration). Standardized procedures would allow easier comparison between different ENMs and forms of the same ENM, enable grouping and read-across between nanoforms, as well as the production of more reliable data that can be implemented in geochemical models. International regulatory bodies, such as ECHA, the Organization for Economic Cooperation and Development (OECD) and the International Organization for Standardization (ISO), have argued that these points should be addressed [31]. To date, several methods have been applied for dissolution testing of ENMs [16]. However, there is no standardized OECD guideline for dissolution testing of ENMs other than the guidance in OECD GD 318 [32,33]. The OECD TG 105 [34] has been evaluated as not applicable for ENMs [35] and OECD GD 29 has been identified as being in principle applicable with some adjustments for determining solubility [36,37]. Nanomaterial-specific OECD test guidelines for dissolution rate testing in environmental media would thus be beneficial for generating data that are regulatory relevant and reliable [38]. 

Generally, dissolution-testing methods can be divided into static batch and continuous flow/flow-through approaches. Batch testing leads to a direct measure of the ENM solubility in a specific aqueous medium. Moreover, when using ultra-trace analytical techniques, it is possible to measure very low ion concentrations. However, it has several limitations, especially when dealing with particles that are rapidly dissolving. For instance, the ultra-centrifugation or centrifugal ultrafiltration of rapidly dissolving particles can lead to an overestimation of the dissolution, because of the dissolution of the particles during the centrifugation process. To the opposite, the dissolved fraction could be underestimated using filtration techniques if the dissolved ions are adsorbed to the ultrafiltration membrane or the walls of the filtration device. Furthermore, batch experiments are performed under static conditions (i.e., limited supply of exposure medium) and may lead to a change of the medium composition, due to ENMs dissolution and/or the re-precipitation of secondary species. The challenges associated with the batch systems can be overcome by using a flow-through/continuous flow setup. The continuous flow dissolution systems are considered to be more appropriate for dissolution rate measurements and present a more environmentally realistic system. It allows for working with high liquid-to-solid ratios (realistic high dilution of the ENM), and when using appropriate analytical instrumentation, it allows for the detection of low levels of dissolved species. Because of a constant and almost unlimited provision of the medium, the back reactions are limited and low electrolyte concentrations in the exposure medium can be used to mimic the chemical composition of natural waters. These results can finally serve as inputs into environmental fate modeling. The flow-through setup has been described in ISO TR 19057 [39]. For instance, Koltermann-Juelly et al. [40] studied the dissolution rates of 24 types of nanomaterials in phagolysosomal simulant fluids, and Bove et al. [31] investigated dissolution of Ag NPs in an in vitro assay simulating conditions which likely occur in human digestion. More recently, Keller et al. [41] applied continuous flow systems to study the dissolution of BaSO_4_ in phagolysosomal and lung lining fluids. These studies demonstrated the advantage of using continuous flow rather than static incubations to investigate and compare the dissolution rates of ENMs. Nevertheless, when adapted to simulate ENMs dissolution in specific biological media, these studies applied relatively low flow rates and high loadings of ENMs, which resemble elevated solid-to-liquid ratios and would not be translatable to environmentally relevant conditions.

Here, we aim to provide guidance and recommendations for nanomaterials dissolution testing and dissolution rates determination under environmentally relevant conditions. Based on a survey of surface water hydrochemistry, we outline the environmental concentration ranges of key parameters to be considered in studying ENMs dissolution in natural aquatic environments. We present the proof of concept of a continuous flow setup suitable to investigate dissolution kinetics at low ENMs concentrations, relevant for natural environments. The results obtained on Ag NPs exposed to artificial waters demonstrate the suitability of the continuous flow dissolution method to determine dissolution rates of ENMs under non-equilibrium environmentally relevant conditions. This work will aid in the standardization of a continuous flow dissolution approach adapted to study ENMs dissolution kinetics in natural aquatic environments, and supports the ongoing development towards OECD dissolution guidelines.

## 2. Materials and Methods

### 2.1. Continuous Flow Testing 

**Figure 1:**
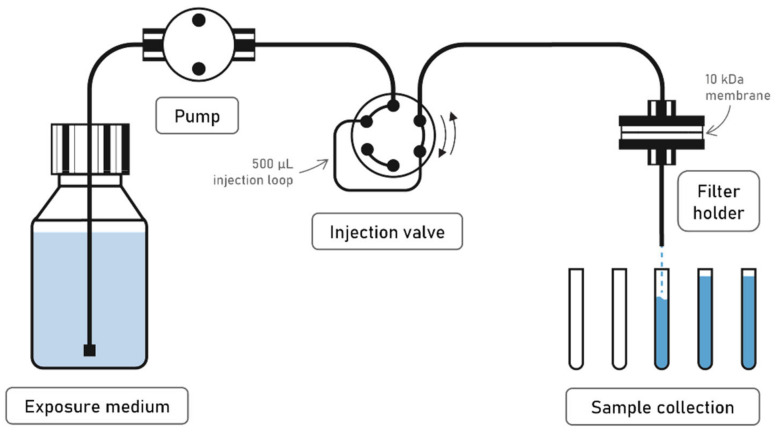
Representation of the setup used for the flow-through dissolution experiments.

Our experimental setup consisted of one 10 kDa Hydrosart^®^ regenerated cellulose membrane of 25 mm diameter (Sartorius Stedim Biotech GmbH, Goettingen, Germany) enclosed into a metal-free polyether ether ketone (PEEK) filter holder (Wyatt Technology Europe GmbH, Dernbach, Germany). After deposition of the NPs onto the membrane, a high-performance liquid chromatography (HPLC)-type piston pump (Postnova Analytics GmbH, Landsberg am Lech, Germany) delivered the continuous flow of medium into the ultrafiltration cell at rates between 0.2 to a few mL.min^−1^ (Figure 1). The exposed solution was then collected at the outlet using an auto-sampler and analyzed for dissolved elemental concentrations. This setup allows the direct separation between the nanoparticulate and dissolved fractions, which are defined by the nominal cut-off of the ultrafiltration membrane, here equal to 10 kDa (10.000 g.mol^−1^), which corresponds to a spherical particle of ~3 nm diameter. Nanoparticles are deposited onto the membrane filter through the injection of a NPs dispersion. The number of nanoparticles deposited at the surface of the membrane is controlled by the concentration of the NPs suspension and the volume injected. In contrast to previous continuous flow dissolution testing, where milligrams of NPs powder are loaded between two membranes, such a method allows to inject smaller amount of ENMs and to have a visible homogeneous coverage of ENMs at the surface of the membrane [10,40]. In particular, a PTFE tube was used as an injection loop for all experiments, in order to inject a volume of 500 µL of the NPs dispersion. This injection loop was connected to the flow-through setup with an injection valve (Figure 1). At the beginning of the experiment, the flow-through system was started in order to let the eluent solution flush the whole system. After a few minutes, the injection loop was connected to the system in order to inject the Ag NP dispersion.

### 2.2. Survey of Surface Water Hydrochemistry 

In order to define a media composition more representative of surface aquatic environments, a survey on selected parameters known to influence the dissolution of ENMs was performed for several river systems. Specifically, pH, Ca^2+^, Mg^2+^, dissolved organic carbon (DOC), orthophosphates, and conductivity were chosen as key factors [21,22,23,42,43,44]. Data for the Danube River were extracted from the TransNational Monitoring Network (TNMN) dataset of the International Commission for the Protection of the Danube River (ICPDR) database [45]. For the Rhine river, data were extracted from the FGG Rhein database for two measuring stations, Karlsruhe and Bad Honnef, located along the Rhine main tributary [46]. Data for the Elbe River were obtained from the specialized information system (FIS) of the FGG Elbe database which have been collected at important measuring stations in the area of the Elbe catchment within the national measuring programs [47]. For these databases, values from 2015 to 2017 were extracted, corresponding to specific sampling locations and times [45,46,47]. In addition, data of the same parameters were extracted from the Forum of European Geological Surveys (FOREGS) [48] and the European Environment Agency (EEA) [49] databases in order to represent a large and global pool of European surface waters. From these data sets, minimum and maximum values, and median, first (Q1), and third (Q3) quartiles were calculated and plotted. 

### 2.3. Experimental Conditions 

Dissolution tests were performed using spherical 80 nm citrate-coated Ag NPs (NanoXact^TM^) obtained from Nanocomposix, San Diego, CA, USA (JRD0035). In order to assess the feasibility of the flow-through dissolution method, a set of experiments were performed at pH 5 with 10 mM NaNO_3_ (Merck, Darmstadt, Germany) as exposure medium. These experiments were performed in order (1) to define optimal experimental parameters for the test and (2) to investigate the influence of extrinsic parameters (i.e., injection velocity, flow rate and ENMs loading). Table 1 summarizes the experimental conditions tested for the dissolution experiments. Ag_0.2mL/min_2.2µg_NaNO_3_ and Ag_0.2mL/min_8.2µg_NaNO_3_ were performed with 2.2 and 8.2 µg of Ag NPs loaded onto the filter, corresponding to 2.99 % and 0.91 % of the filter area covered by Ag NPs, respectively. For these experiments, the injection flow rate was set to 1 mL.min^−1^ during 1 h. After injection, the flow rate was reduced to 0.2 mL.min^−1^ until the end of the experiment. Ag_0.5mL/min_8.2µg_NaNO_3_ was performed with 8.2 µg of Ag NPs loaded onto the membrane at a flow rate of 0.5 mL.min^−1^, constant during all the time of the experiment. Ag_0.2mL/min_2.2µg_NaNO_3_, Ag_0.2mL/min_8.2µg_NaNO_3_ and Ag_0.5mL/min_8.2µg_NaNO_3_ were performed in duplicates. All other experiments were performed with an Ag NPs loading of 8.2 µg and a constant flow rate of 0.5 mL.min^−1^. For the latter, 2 mM Ca(NO_3_)_2_ and 0.5 mM MgSO_4_ solutions (Merck, Darmstadt, Germany) were used in order to have a Ca^2+^/Mg^2+^ ratio of 4:1, as recommended by the OECD guideline 318 [33]. To simulate acidic surface waters (ASW), Ag_0.5mL/min_8.2µg_ASW was performed at pH 5. The pH of the eluent was adjusted using 0.2 M HNO_3_. To simulate near-neutral surface waters (NSW), Ag_0.5mL/min_8.2µg_NSW was performed at pH 7.5 by adding 5 mM NaHCO_3_^−^ to the Ca(NO_3_)_2_-MgSO_4_ solution, acting as pH buffer. As part of this work, no DOC and orthophosphate were included, the aim being to show a proof of concept of the continuous flow dissolution setup instead of discussing the impact of ligands on the dissolution kinetic of Ag NPs.

### 2.4. Samples Measurement and Data Treatment

For all experiments, samples were taken at 5, 10, 15, or 20 min intervals directly into 15 mL PP vials, acidified with 20 µL of 65%HNO_3_ (AnalaR Normapur^®^, VWR, Austria), and analyzed by inductively coupled plasma-mass spectrometry (Agilent 7900 ICP-MS, Agilent Technologies, Tokyo, Japan) for dissolved Ag concentrations. The ICP-MS was equipped with a Ni cone, a MicroMist nebulizer and a scott-type double pass spray chamber. It was operated on standard mode, under continuous Ar gas flow (UHP, Nebulizer gas flow rate at 0.8 L.min^−1^; Dilution gas flow rate of 0.4 L.min^−1^). The two stable isotopes ^107^Ag and ^109^Ag were measured for higher accuracy, and Rhodium (Rh, solution of 10 µg.L^−1^) was used as internal standard. The ICP-MS was calibrated with dissolved Ag standards ranging from 5 ng.L^−1^ to 50 μg.L^−1^ prepared from a single-element Ag standard (1000 μg.mL^−1^; CGAG1−125 ml, Inorganic Ventures, Christiansburg, VA, USA) diluted with 2% HNO_3_. The acid blanks for all measurement times show an averaged limit of detection (LOD; 3× standard deviation + mean) of 0.05 μg.mL^−1^and an averaged limit of quantification (LOQ; 10× standard deviation + mean) of 0.12 μg.mL^−1^. Measurements were carried out in triplicates.

Experiment Ag_0.2mL/min_2.2µg_NaNO_3_ was performed for 5 h. Ag_0.2mL/min_8.2µg_NaNO_3_, Ag_0.5mL/min_8.2µg_NaNO_3_, Ag_0.5mL/min_8.2µg_ASW and Ag_0.5mL/min_8.2µg_NSW were performed for 8 h. For each experiment, apparent dissolution rates *k* were calculated from the outflow concentrations using Equation (1):

*k* = [Ag]_outlet_ × F/SA (1)

where *k* in µg.s^−1^.m^−2^, [Ag]_outlet_ the Ag concentration measured at the outlet in µg.mL^−1^, F the flow rate of the eluent in mL.s^−1^, and SA the combined surface area of the NPs deposited on the membrane in m^2^. SA was determined considering the surface area of an 80 nm diameter spherical NP, NP_SA_ and the total number of NPs deposited onto the membrane, NPs_membrane_ (SA = NP_SA_ × NPs_membrane_). 

## 3. Results and Discussion

### 3.1. Hydrochemical Conditions to Investigate ENMs Dissolution in Natural Aquatic Media

**Figure 2:**
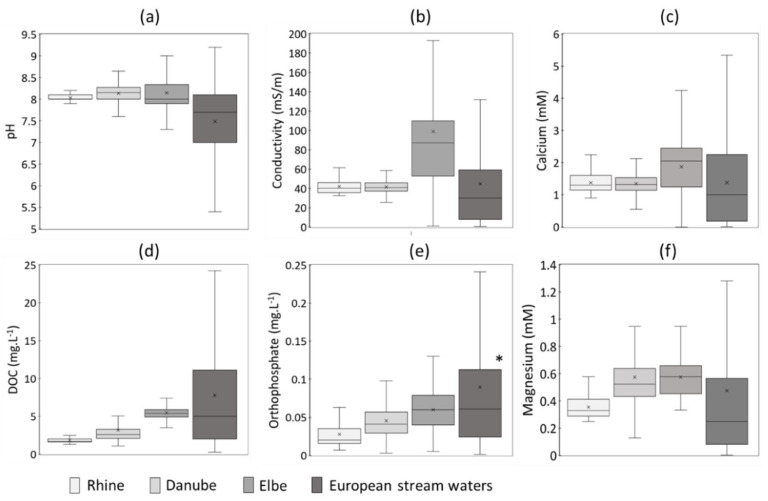
Boxplots illustrating the range values of (**a**) pH, (**b**) conductivity, (**c**) calcium concentration, (**d**) DOC, (**e**) orthophosphate and (**f**) magnesium concentrations in specific and a pool of surface waters. Data from 2015 to 2017 were obtained from The International Commission for the Protection of the Danube River (ICPDR) [45], the River Basin Communities of Rhine [46] and the Elbe Data Information System (FIS) of the River Basin Community [47] for the Danube, Rhine, and Elbe rivers, respectively. Data from the Rhine River were obtained at the locations Bad Honnef and Karlsruhe. The FOREGS (Forum of European Geological Surveys) Geochemical database [48] and the European Environment Agency (EEA) database were used to represent European stream waters. * Data obtained from the EEA database “Waterbase-Water Quality” [49].

Studies on solubility and dissolution kinetics are usually performed using deionized water or simple background electrolyte solutions (e.g., NaCl, NaNO_3_ or Ca(NO_3_)_2_) for specific pH values [15,16]. However, the lack of complexity in the exposure media is not suited to mimic ENMs dissolution in natural waters, which depends on an interplay between intrinsic properties and extrinsic environmental parameters, unique to each environment. Few variations are observed within one river system, such as the Danube and Rhine rivers, and to a larger extent for the Elbe River. In contrast, the FOREGS database, issued from the Geochemical Atlas of Europe [48], presents a wide range of values (Figure 2) covering the diversity of various catchments on the continent. The FOREGS database is, to date, the most relevant database to define media composition, mimicking a large pool of natural surface waters, from alkaline rivers such as Danube and Rhine (Figure 2) to more acidic waters mostly present in base-poor buffering capacity regions and/or organic-rich acid buffering regions. However, it does not provide data on all chemical components, such as orthophosphates. For this study, orthophosphate concentrations that are representative of a pool of rivers were obtained from the European Environment Agency (EEA) database “Waterbase-Water Quality” [49].

As recommended by OECD GD318 for the testing of nanomaterials dispersion stability [32], exposure media harboring low concentrations of the major ions reported in surfaces waters, Ca^2+^ and Mg^2+^, would allow a better simulation of the ionic background of surface waters [48]. In addition to standard tests performed using 10 mM Na(NO_3_), we thus propose to use 2 mM of Ca(NO_3_)_2_ and 0.5 mM of MgSO_4_ to mimic a more realistic scenario of natural waters. Such values are based on Ca^2+^ and Mg^2+^ concentrations reported by the FOREGS database, with average concentration of 1.4 mM and 0.5 mM for Ca^2+^ and Mg^2+^, respectively [48]. To simulate alkaline surface waters (pH 7–8), 2 to 5 mM HCO_3_^−^ should be added to the exposure media. 

For all databases, phosphate concentrations are significant, with values ranging between 0.001 to 0.190 mg.L^−1^ (Figure 2). Regarding the expected concentrations of ENMs into aquatic environments, for example a maximum of 150 ng.L^−1^ for ZnO NPs [5], phosphate can thus play an important role by initiating transformation or formation of lower soluble metal-phosphate coatings. Similarly, NOM in surface waters (Figure 2) might play an important role, enhancing or inhibiting dissolution of ENMs [21,22,50]. Thus, for a more realistic scenario, test medium should also include NOM and inorganic phosphate. We should, however, point out the importance of separating two pivotal processes in environmental aquatic media: dissolution and chemical transformation. Testing using a complex aquatic chemistry (i.e., sulfide, Cl^−^, PO_4_^3−^, HCO_3_^−^, DOC) might trigger ENM transformations into less soluble phases [51,52,53] and affect the direct evaluation of the solubility and dissolution rate. Determination of the dissolution rates in a medium that represents natural conditions but does not induce other reactions is then necessary. In this regard, precipitation of secondary species must be preliminarily predicted using thermodynamic modeling. 

### 3.2. Dissolution of Ag NPs in Simple Background Electrolyte: Validation of the Continuous Flow System for Low Ag NPs Loadings

**Figure 3:**
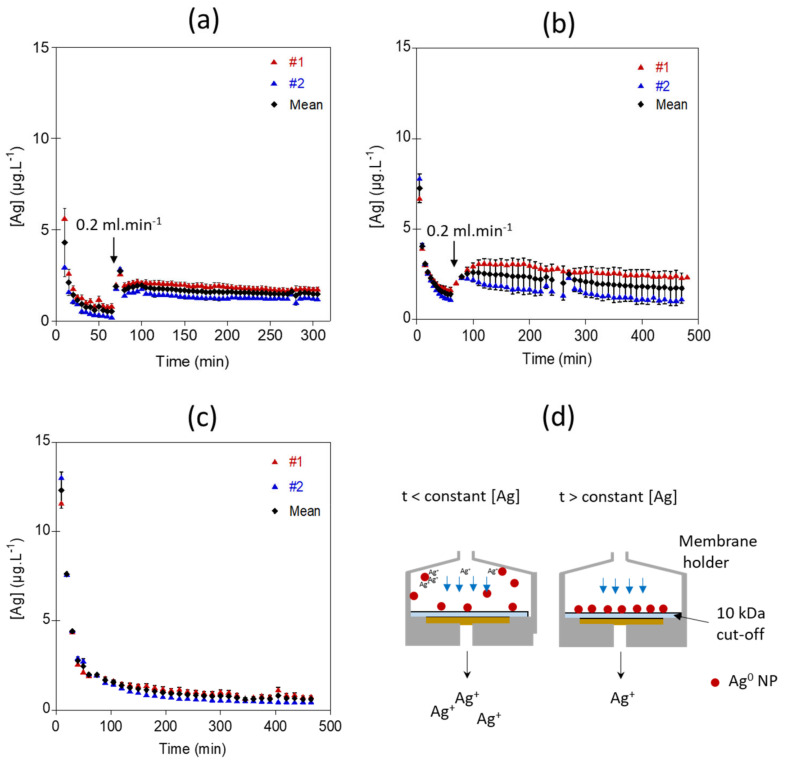
Ag concentrations measured at the outlet of the flow-through setup for (**a**) 500 µL of a 4.3 mg.L^−1^ Ag NPs suspension injected with a flow rate of 1 mL.min^−1^ for 1 h and set to 0.2 mL.min^−1^ for the rest of the experiment, Ag_0.2mL/min_2.2µg_NaNO_3_ experiment (**b**) for 500 µL of a 16.4 mg.L^−1^ Ag NPs suspension injected with a flow rate of 1 mL.min^−1^ for 1 h and set to 0.2 mL.min^−1^ for the rest of the experiment, Ag_0.2mL/min_8.2µg_NaNO_3_ experiment and (**c**) for 500 µL of a 16.4 mg.L^−1^ Ag NPs suspension injected with a flow rate set to 0.5 mL.min^−1^ during all the time of the experiment, Ag_0.5mL/min_8.2µg_NaNO_3_ experiment. (**d**) Conceptual illustration of the behavior of Ag NPs in the filter holder during the flow-through experiment.

For all experiments performed at pH 5 with 10 mM NaNO_3_ as the eluent, higher dissolved Ag concentrations were measured at the beginning of the exposure experiments (Figure 3). During the first minutes of the exposure, the dissolution behavior of the Ag NPs might be governed by the positioning of the particles in the filter holder. Since the particles are not immediately fixed onto the membrane, they may remain dispersed in the filter holder’s dead volume above the membrane, increasing the contact time with the eluent and consequently the Ag concentration. Similarly, the increase in Ag concentrations after 60 min exposure for the experiment Ag_0.2mL/min_2.2µg_NaNO_3_ and Ag_0.2mL/min_8.2µg_NaNO_3_ correspond to an increase in contact time with the eluant due to the decrease in the flow rate from 1 mL.min^−1^ to 0.2 mL.min^−1^. The presence of dissolved Ag^+^ in the injected Ag suspension and sorbed Ag^+^ at the surface of the NPs could also explain higher Ag concentrations at the beginning of the experiments.

**Figure 4:**
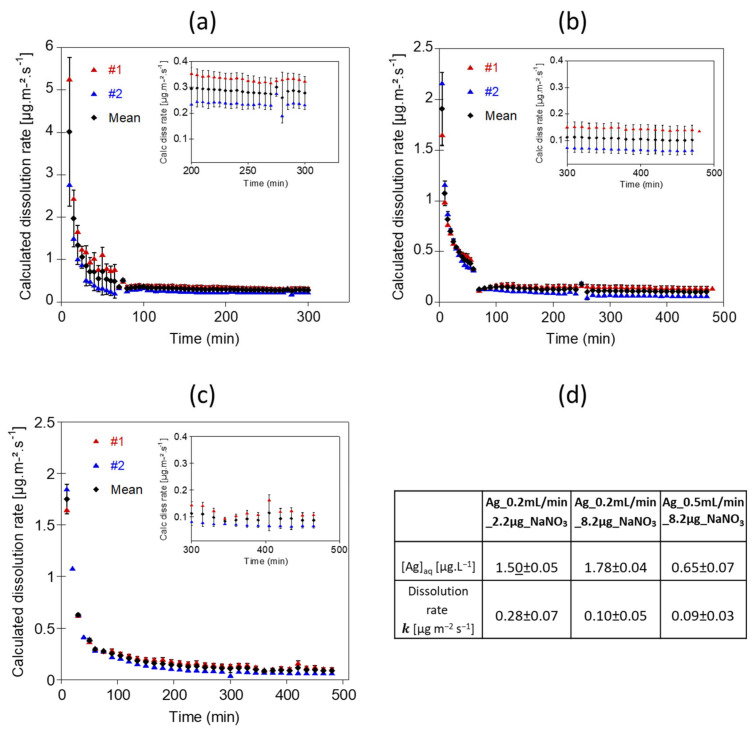
Dissolution rates of 80 nm Ag NPs in 10 mM NaNO_3_ at pH 5, for different particles loadings and flow rates. (**a**) Dissolution rates obtained for the Ag_0.2mL/min_2.2µg_NaNO_3_ experiment corresponding to 2.2 µg Ag NPs exposed at a flow rate of 1 mL.min^−1^ for 1 h and 0.2 mL.min^−1^ for the rest of the experiment. (**b**) Dissolution rates obtained for Ag_0.2mL/min_8.2µg_NaNO_3_ experiment corresponding to 8.2 µg Ag NPs exposed at a flow rate of 1 mL.min^−1^ for 1 h and 0.2 mL.min^−1^ for the rest of the experiment. (**c**) Dissolution rates obtained for Ag_0.5 mL/min_8.2µg_NaNO_3_ experiment corresponding to 8.2 µg Ag NPs exposed at a flow rate of 0.5 mL.min^−1^. (**d**) Table showing the average [Ag] concentrations and the averaged dissolution rates calculated on the steady state ranges.

Once the particles are deposited onto the membrane and are exposed to a continuous and unchanged flow rate, outflow Ag concentration becomes stable (Figure 3). The apparent dissolution rates calculated from the Ag concentrations (Figure 4) reached a steady state after 225 min for Ag_0.2mL/min_2.2µg_NaNO_3_, 400 min for Ag_0.2mL/min_8.2µg_NaNO_3_, and 345 min for Ag_0.5mL/min_8.2µg_NaNO_3_. The average Ag concentrations and averaged dissolution rates were calculated on the steady state range. Experiments performed with 8.2 µg of Ag NPs loaded onto the membrane at two different flow rates show similar averaged dissolution rates (k = 0.10 ± 0.002 µg.m^−2^.s^−1^ and k = 0.093 ± 0.009 µg.m^−2^.s^−1^ for Ag_0.2mL/min_8.2µg_NaNO_3_, and Ag_0.5mL/min_8.2µg_NaNO_3_, respectively). Nevertheless, for the experiment performed at a flow rate of 0.2 mL.min^−1^, the averaged Ag concentration at the outlet was 2.7 times higher, due to a longer contact time of the eluent with Ag NPs (Figure 3 and Figure 4d). Theoretically, lower flow rates would allow more contact time between the eluent and the surface of the NPs, leading to higher Ag concentrations in the reaction zone and result in a larger diffusion boundary layer, both limiting the dissolution process. The study of Keller et al. [54] illustrates well the effect of the flow-rate on the dissolution kinetics of nanomaterials such as BaSO_4_ NPs, by performing long term experiments with higher NPs loadings and flow rate ramping between 0.1 and 3.0 mL.h^−1^. Dissolution rates are not much different between the experiments Ag_0.2mL/min_8.2µg_NaNO_3_, and Ag_0.5mL/min_8.2µg_NaNO_3_. However, it is likely that at higher flow rates a more pronounced difference will be observed. However, for highly soluble ENMs such as CuO and ZnO NPs, the influence of the flow rate on dissolution might be reduced [54].

Ag NPs loading appear to influence significantly the dissolution rate of Ag NPs when applying the same 0.2 mL.min^−1^ flow (experiments Ag_0.2mL/min_2.2µg_NaNO_3_ and Ag_0.2mL/min_8.2µg_NaNO_3_, Figure 4d). The dissolution rate was more than two times higher for the experiment performed with 2.2 µg of Ag NPs (Ag_0.2mL/min_2.2µg_NaNO_3_, k = 0.28 ± 0.07 µg.m^−2^.s^−1^) compared with the experiment performed with 8.2 µg of Ag NPs (Ag_0.2mL/min_8.2µg_NaNO_3_, k = 0.10 ± 0.05 µg.m^−2^.s^−1^). A high particle loading increases the dissolved ion concentration in the vicinity of the particle surfaces, limiting their dissolution. To avoid local saturation around the nanoparticles, a higher flow rate might be used. Nevertheless, the dissolution rates determined at a flow rate of 0.5 and 0.2 mL.min^−1^ are similar (k = 0.09 ± 0.03 compared with k = 0.10 ± 0.05 µg.m^−2^.s^−1^, respectively, both at 8.2 µg loading and with NaNO_3_). This indicates that the dissolution rate of Ag NPs depends on the initial Ag NPs concentration. Concentration-dependent dissolution of Ag NPs was already reported by several studies, showing higher dissolution rates for lower Ag NPs concentrations [55,56]. Keller et al. [54] also highlighted the influence of the initial NPs loading on the dissolution rate for BaSO_4_, CuO, ZnO and TiO_2_ NPs. Such findings emphasize the importance to investigate ENMs dissolution at environmentally relevant ENMs concentrations. Indeed, performing flow through dissolution experiments using high initial NPs loading may result in wrong assessments of the dissolution rate of an ENM in natural aquatic systems. We may also hypothesize that a larger amount of Ag NPs injected does not result in the deposition of Ag NPs as a monolayer.

The disparity between the dissolution rates calculated for each experiment performed using the same Ag NPs and the same exposure medium demonstrated that the dissolution kinetic of Ag NPs is dependent on the initial NPs concentration. The flow rate is also an important parameter to adjust for the success of the test and an appropriate representation of the system to mimic. A low flow rate allows for longer interaction between the medium and the ENMs, which would lead to a more reliable measurement of the dissolved fraction at the outlet. Suitable for ENMs with low solubility, it may also result in local saturation and underestimation of the dissolution rate. Experimental parameters (i.e., flow rate, exposure time and ENMs loading) need to be adjusted to the environmental conditions we want to mimic and to the solubility property of the studied ENM, keeping the feasibility of the test. Based on the results obtained through these tests, we recommend performing continuous flow dissolution experiments at a flow rate between 0.5 and 1 mL.min^−1^, sufficient to reach a steady-state after 5–6 h of exposure, and allowing robust determination of the outlet dissolved concentrations while reducing potential saturation effects. 

### 3.3. Dissolution Rate of Ag NPs in Artificial Surface Waters

**Figure 5:**
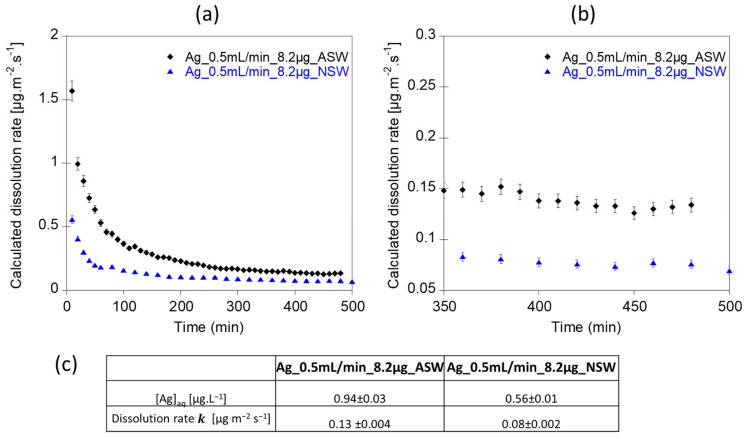
(**a**,**b**) Dissolution rates of 80 nm Ag NPs in environmental aqueous media. (**c**) Table showing the average [Ag] concentrations and the averaged dissolution rates calculated on the steady state ranges. Ag_0.5mL/min_8.2µg_ASW was performed at pH 5 with 2 mM Ca(NO_3_)_2_ and 0.5 mM MgSO_4_ as exposure medium. Ag_0.5mL/min_8.2µg_NSW was performed at pH 7.5 with 2 mM Ca(NO_3_)_2_, 0.5 mM MgSO_4_ and 5 mM of NaHCO_3_^−^ as exposure medium. Error bars correspond to the incertitude of Ag concentrations measured by ICP-MS.

Continuous flow experiments were also performed using artificial waters intended to mimic acidic streams (2 mM Ca(NO_3_)_2_ and 0.5 mM MgSO_4_ solution at pH 5) and near-neutral/alkaline surface waters (2 mM Ca(NO_3_)_2_, 0.5 mM MgSO_4_ and 5mM NaHCO_3_^−^ solution at pH 7.5). In both experiments, the steady-state plateau was reached after 400 min (Figure 5a,b). The averaged dissolution rate of Ag NPs calculated for the near-neutral artificial water exposure experiment was relatively low (Ag_0.5mL/min_8.2µg_NSW, k = 0.08 µg.m^−2^.s^−1^). For this latter experiment, the averaged concentration of Ag measured in the eluant ([Ag] = 0.56 ± 0.01 µg.L^−1^) was higher than the LOD (0.05 µg.L^−1^) and LOQ (0.12 µg.L^−1^) values determined for the ICP-MS. However, for the lower Ag NP dissolution rate, lower Ag concentrations at the outlet might impact the relevance and significance of the test, for example, if dissolved Ag concentrations are too low to be accurately measured by ICP-MS (Figure 5c). In such case, lower dilution factors and/or a lower flow rate might thus be suitable.

At a lower pH, the averaged Ag dissolution rate was higher (Ag_0.5mL/min_8.2µg_ASW, k = 0.13 µg.m^−2^.s^−1^) than at pH 7.5 (Ag_0.5mL/min_8.2µg_NSW, k = 0.08 µg.m^−2^.s^−1^). This trend is consistent with previous work showing an increase in Ag NPs dissolution in acidic media [23]. Using the classic batch experiment, Mitrano et al. [57] investigated the dissolution rate of 100 nm and 60 nm spherical citrate-coated Ag NPs in artificial and natural media. They reported dissolution rate values as logr = −11.72 and −12.23 mol.cm^−2^.s^−1^ in deionized water (pH = 6.7), logr = −12.14 and −12.71 mol.cm^−2^.s^−1^ in a natural surface water (pH = 7.3) and logr = −12.46 mol.cm^−2^.s^−1^ in deionized water containing 2 mg.L^−1^ of DOC (pH = 4.8). Such values are close to the values obtained in this study, with log *k* ranging between −12.58 and −13.13 mol.cm^−2^.s^−1^ for Ag_0.2mL/min_2.2µg_NaNO_3_ and Ag_0.5mL/min_8.2µg_NSW, respectively.

## 4. Conclusions

It is encouraged to develop standardized experimental procedures into OECD test guidelines to evaluate the dissolution rate of nanomaterials in natural aquatic environments [32]. Flow-through/continuous flow dissolution experiments are well suited to study dissolution kinetics of NPs and to systematically investigate NPs dissolution rates under environmentally relevant conditions. The main advantages are (1) a continuous supply of exposure media, (2) the possibility to expose small amounts of NPs (low solid/liquid ratio), and (3) the instantaneous separation between the dissolved and the solid fractions. For ENMs of very high or very low solubility, the flow-through testing can be easily adjusted according to the fast depletion of the material in the one, and too small outlet concentrations in the other case (i.e., NPs loading, flow rate). In contrast to recent studies investigating dissolution of ENMs in physiological fluids using a continuous flow system [40,41,54], our experimental approach uses the injection of ENMs dispersions which allows to load significantly lower amount of ENMs, relevant to mimic realistic natural environmental settings [5,58]. Our results demonstrated the feasibility to run flow-through dissolution tests for ENMs loadings down to the microgram range using environmentally relevant exposure media. The influence of the flow rate on the thickness of the diffusional boundary layer, the dissolution rate [54], and the local saturation at the vicinity of the nanoparticles should be further investigated. In particular, local saturation at the vicinity of the nanoparticles may occur in natural settings, for example, in water-saturated soils and sediments where pore waters may have longer residence time. In such specific aquatic compartments, it is relevant to determine various dissolution rates from long term experiments with various flow rates. In addition, establishing a clear recommendation of media compositions that would represent a wide range of natural waters, covering the dissolution-relevant species, would bring the tests closer to environmental realism. Here, it must be differentiated between standard testing for the comparison of materials regarding only their dissolution behavior, and tests, which aim to observe realistic environmental behavior including possible ENMs transformations. In the first case, a controlled pH and a simple inert background electrolyte would be suitable, whereas in the latter case, a more complex water chemistry including phosphate, sulfate, chloride, sulfide, and NOM (and others) would be chosen. In summary, there is a growing need for standardizing and implementing continuous flow dissolution testing in test guidelines to address a more realistic scenario of ENMs in natural aquatic systems. This would improve reproducibility and comparability of results for the wide diversity of pristine and transformed ENMs and allow their registration with international regulatory bodies.

## Figures and Tables

**Table 1:** Experimental parameters and media composition used to perform the flow-through experiments.

	Ag_0.2mL/min _2.2µg_ NaNO_3_	Ag_0.2mL/min _8.2µg_ NaNO_3_	Ag_0.5mL/min _8.2µg_ NaNO_3_	Ag_0.5mL/min _8.2µg_ ASW	Ag_0.5mL/min _8.2µg_ NSW
Setup parameters					
[Ag NPs]^1^ [mg.L^−1^]	4.3	16.4	16.4	16.4	16.4
Volume injected [µL]	500	500	500	500	500
Ag NPs loading [µg]	2.2	8.2	8.2	8.2	8.2
Injection time [h]	1	1	-	-	-
Injection flow rate [mL.min^−1^]	1	1	-	-	-
Flow rate experiment [mL.min^−1^]	0.2	0.2	0.5	0.5	0.5
Exposure media					
pH	5	5	5	5	7.5
NaNO_3_ [mM]	10	10	10	-	-
Ca(NO_3_)_2_ [mM]	-	-	-	2	2
MgSO_4_ [mM]	-			0.5	0.5
HCO_3_^−^ [mM]	-	-	-	-	5

^1^ the Ag NPs concentration injected was determined by acid digestion of 500 µL of the 20 mg.L^−1^ Ag NPs stock solution. For Ag_0.2mL/min_2.2µg_NaNO_3_, the volume of the 20 mg.L^−1^ Ag NPs stock solution used to prepare the 5 mg.L^−1^ solution was acid digested and Ag NPs concentration was thus assume from the dilution factor.
